# Head and neck imaging manifestations in COVID-19: collective experience of 17 months during 2nd wave of the pandemic

**DOI:** 10.1186/s43055-023-00968-4

**Published:** 2023-01-17

**Authors:** Balasubramanian Gurumurthy, Rudresh Hiremath, Anupama Chandrappa, Rakesh Chowkalli Veerabhadrappa, Divya Vishwanatha Kini, Sai Siddartha Kosinepalli

**Affiliations:** 1 Department of Radiology, JSS Hospital, JSS Academy of Higher Education and Research, Ramachandra Agrahara, Mysore, Karnataka 570004 India; 2 Department of Speech and Hearing, Manipal College of Health Professions, Manipal, India

**Keywords:** COVID-19, Head and neck, Sinusitis, Dacryoadenitis, Otitis media

## Abstract

**Background:**

COVID-19 is well known to result in pulmonary and multiple extra-pulmonary manifestations. Among them, head and neck manifestations were commonly recognized in the 2nd wave of the pandemic. With the growing global COVID-19 burden, imaging is of utmost importance in diagnosing the disease and its related complications. The study aims to enumerate the various head and neck manifestations and their complications in COVID-19. Additionally, in sinusitis patients, the invasion was correlated with the neutrophil–lymphocyte ratio (NLR).

**Results:**

A cross-sectional observational study in which total of 78 COVID-19 cases that underwent head and neck imaging were retrospectively evaluated. The cohort included 52 males (66.7%) and 26 females (33.3%) with a mean age of 46.19 years (median = 49.0, SD = 16.47). The various head and neck manifestations included non invasive rhinosinusitis (*n* = 48), invasive sinusitis and its complications (*n* = 25), nasal septal abscess (*n* = 1), dacryoadenitis (*n* = 1), pre-septal and post-septal orbital cellulitis and its complications (*n* = 13), otitis media, mastoiditis and its complications (*n* = 6), parotitis (*n* = 2), neck vessel thrombosis (*n* = 2) and cervical lymphadenopathy (*n* = 3). An increase in the invasive nature of sinusitis was demonstrated among patients with comorbidities and elevated NLR.

**Conclusions:**

Early diagnosis and management of head and neck manifestations of COVID-19 are aided by prompt imaging. It is imperative that we are armed with the knowledge of various head and neck manifestations and how they may bear semblance to other pathologies for us to ensure COVID as a differential, especially in the background of known infection.

## Background

Coronavirus disease 2019 (COVID-19) has spread globally ever since it was declared a pandemic by the World Health Organization on March 11, 2020 [1]. COVID-19 is well known to result in pulmonary and multiple extra-pulmonary manifestations [1]. Among the extra-pulmonary manifestations, head and neck involvement, primarily of the sino-nasal, orbital and otic compartments, were increasingly recognized during the second wave of pandemic [2, 3].

The pandemic of SARS-CoV-2 persists to be tenacious, with new variants emerging and causing a substantial threat to the human population. The second wave of the pandemic in India manifested an unprecedented spike in COVID-19 to be associated with mucormycosis infection, with most patients presenting with sino-nasal and orbital involvement. Apart from a few case reports and studies on fungal invasive sinusitis, the various head and neck imaging manifestations of COVID-19 and their complications are less distinctly described [4–6]. The present study aimed to enumerate the various head and neck manifestations observed at our institute during the second wave of the pandemic over a period of 17 months. The correlation between the presence of invasive sinusitis with neutrophil–lymphocyte ratio (NLR) was also studied.

## Methods

### Study design and data collection

This was a single-center cross-sectional retrospective observational study conducted for a period of 17 months between March 2021 and August 2022. The study included reverse-transcriptase polymerase chain reaction (RT-PCR) confirmed COVID-19 disease patients and post-COVID-19 disease recovered patients who presented with head and neck symptoms and underwent dedicated imaging for the same. The CT and MRI cross-sectional images were evaluated using the institutional picture archiving and communication systems (PACS) database system to assess the various head and neck manifestations. The Institutional Ethics committee approved this study.

### Selection criteria

The study included active confirmed COVID-19 cases and post-COVID-19 recovered cases that underwent head and neck imaging. The study also included COVID-19 patients with cerebrovascular accidents who underwent Doppler neck ultrasonography. Patients who opted to be discharged against medical advice and patients with history of recurrent rhinosinusitis and otological symptoms were excluded.

### Imaging protocol

CT was performed using a 128-slice MDCT scanner (Ingenuity core 128 v3.5.7.25001; Philips Healthcare) and a 3.0 Tesla MRI machine (Philips Ingenia). Ultrasonography was performed using GE health care Voluson E6 and portable machine GE Health care LogiqE. Based on the clinical presentation, ultrasonography with Doppler studies of the neck, computed tomography of paranasal sinuses and high-resolution computed tomography (HRCT) of temporal bones were performed. Contrast-enhanced MRI brain was subsequently performed when orbital or intracranial involvement was suspected using the following sequences—axial diffusion-weighted imaging (DWI), axial T2W, axial fluid-attenuated inversion recovery (FLAIR), axial T1W, axial gradient recalled echo (GRE), sagittal T1W, coronal T2W, 3D- FLAIR, pre-contrast 3D-T1FS and post-contrast T1FS. Contrast-enhanced MR of orbits was performed for patients presenting with primary ocular symptoms using the following sequences—axial DWI; axial gradient recalled echo (GRE), three planes of T2FS, coronal pre-contrast T1FS, 3D–FLAIR, three planes of post-contrast T1FS.

A total of 40 patients underwent only computed tomography, four cases underwent only non-contrast-enhanced MR imaging, 29 cases underwent both CT and contrast-enhanced MRI, and seven cases underwent ultrasonography.

### Imaging interpretation

The cross-sectional images of CT and MRI were evaluated independently by three radiologists with experience of 16 years, 11 years and six years, respectively. The senior radiologists were blinded to the cohort’s mortality, neutrophil–lymphocyte ratio and comorbidities for the rhinosinusitis cases.

**Statistical analysis**: Raw data were tabulated and analyzed using IBM SPSS software version 28.0.1.1. Among the rhinosinusitis cases, measures of central tendency (mean and median) were calculated, and the data were subjected to a normality test. As the data did not follow the normal distribution, the nonparametric test was carried out. The spearmen’s correlation test was used to check the correlation between the variables (NLR, invasive features, comorbidities and mortality).

## Results

### Demography

Seventy-eight COVID-19 patients that underwent head and neck imaging between March 2021 and August 2022, were retrospectively evaluated. Out of 78 cases, 22 were RT-PCR confirmed cases, and 56 were post-COVID-19 cases.

Descriptive statistics highlighted that the mean age of the cohort was 46.19 years (Median = 49.0, SD = 16.47), with the age group (51–60 years) having the highest number of cases [*n* = 20(25.6%)]. The age distribution is depicted in Table 1. There was a predilection for males [*n* = 52 (66.7%)] when compared to females [*n* = 26 (33.3%)]. The presenting symptoms and various head and neck manifestations are depicted in Tables 2 and 3, respectively.

**Table 1:** Age distribution of the cohort

Age (in years)	Total number (out of 78 cases)	Percentage (%)
0–10	2	2.6
11–20	1	1.3
21–30	11	14.1
31–40	18	23.0
41–50	13	16.7
51–60	20	25.6
61–70	9	11.5
71–80	3	3.9
81–90	1	1.3

**Table 2:** Presenting symptoms of the cohort

Sino-nasal manifestations (out of 73 cases)		Orbital manifestations (out of 14 cases)		Otological manifestations (out of 6 cases)		Others (out of 78 cases)	
Symptoms	Percentage	Symptoms	Percentage	Symptoms	Percentage	Symptoms	Percentage
Headache	19 (26.0%)	Proptosis	6(42.8%)	Otalgia	3(50%)	Fever	25 (32.0%)
Nasal obstruction	26 (35.6%)	Ocular pain	8(57.1%)	Otorrhea	2(33.3%)	Myalgia	4 (5.1%)
Rhinorrhea	10 (13.7%)	Blurring of vision	3(21.4%)	Reduced hearing	2(33.3%)	Cough	6 (7.7%)
Foul smelling nasal discharge	2 (2.7%)	Ophthalmoplegia	3(21.4)			Breathlessness	4 (5.1%)
Epistaxis	2 (2.7%)	Eyelid swelling	8(57.1)			Parotid region swelling	2 (2.5%)
Facial pain	15 (20.5%)	Ocular discharge	1(7.1)			Loss of consciousness	1 (1.2%)
Facial swelling	7 (9.5%)	Diplopia	1(7.1)			Chest pain	1 (1.2%)
Facial discoloration	2 (2.7%)					Altered sensorium	2 (2.5%)
						Neck swelling	3 (3.8%)

**Table 3:** Head and neck manifestations of COVID-19 among the study group

Serial number	Head and neck imaging features	Number of cases	Percentage (out of 78 cases)
1.	Sino-nasal manifestations	73	93.6
2.	Orbital manifestations	14	17.9
3.	Otological manifestations	6	7.6
4.	Salivary glands	2	2.5
5.	Cervical lymph nodes	3	3.8
6.	Neck vessels	2	2.5

### Imaging features

#### Sino-nasal manifestations

Of the 78 cases, 73(93.6%) showed features of rhinosinusitis, and 34 were associated with comorbidities. Among these, maxillary sinuses were most commonly involved [*n* = 63(86.3%)] followed by ethmoidal sinuses [*n* = 52 (71.2%)], sphenoid sinuses [*n* = 38 (52.0%)] and frontal sinuses [*n* = 36 (49.3%)] as depicted in Table 4.

**Table 4:** Sino-nasal and orbital manifestations of the cohort

Sino-nasal Manifestations (out of 73)			Orbital manifestations (out of 14)		
	Number of cases	Percentage		Number of cases	Percentage
Maxillary sinusitis	63	86.3	Dacryoadenitis	1	7.1
Ethmoidal sinusitis	52	71.2	Pre-septal Orbital cellulitis	8	57.1
Sphenoid sinusitis	38	52.0	Post-septal Orbital cellulitis	13	92.8
Frontal sinusitis	36	49.3	Intraconal abscess	2	14.2
Nasal septal abscess	1	1.3	Optic neuritis	5	35.7

**Fig. 1:**
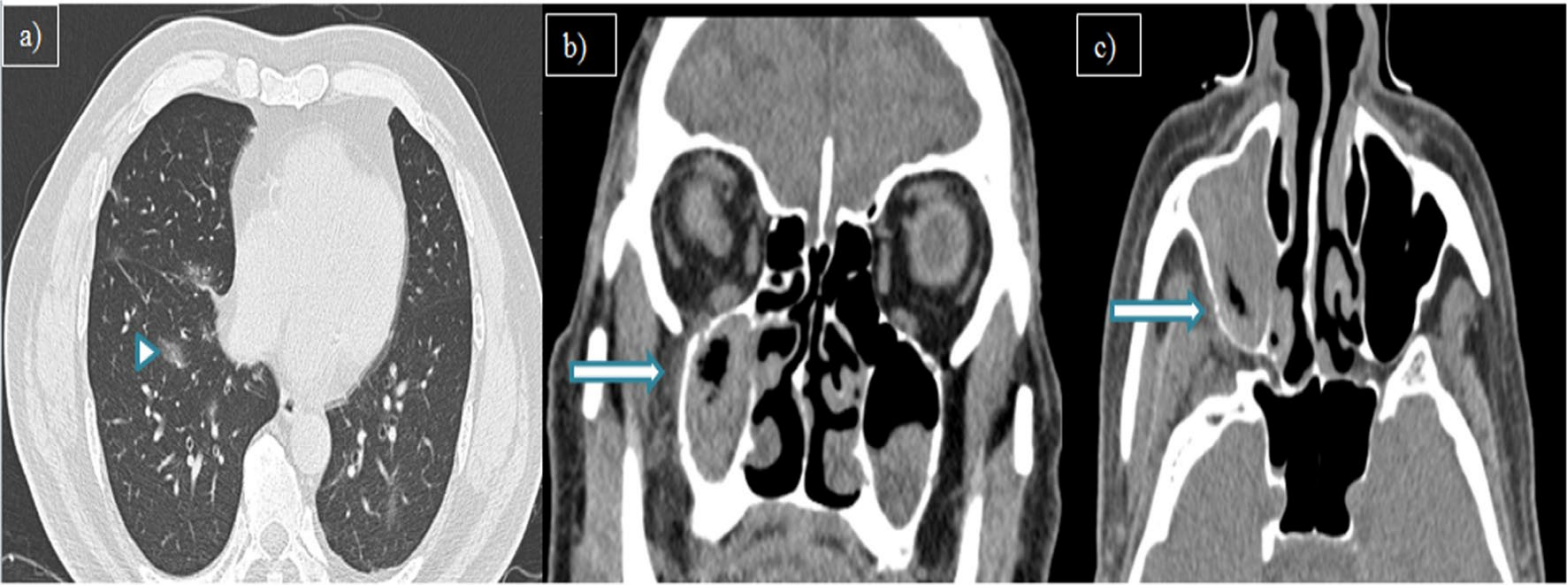
**a** Axial HRCT thorax, **b** Coronal, and **c** Axial CT paranasal sinus images of a 44-year-old male patient with complaints of breathlessness and rhinorrhea showing patchy ground glass opacities in right lower lobe (arrowhead) and bilateral maxillary sinusitis with periantral space fat stranding (arrow)

**Fig. 2:**
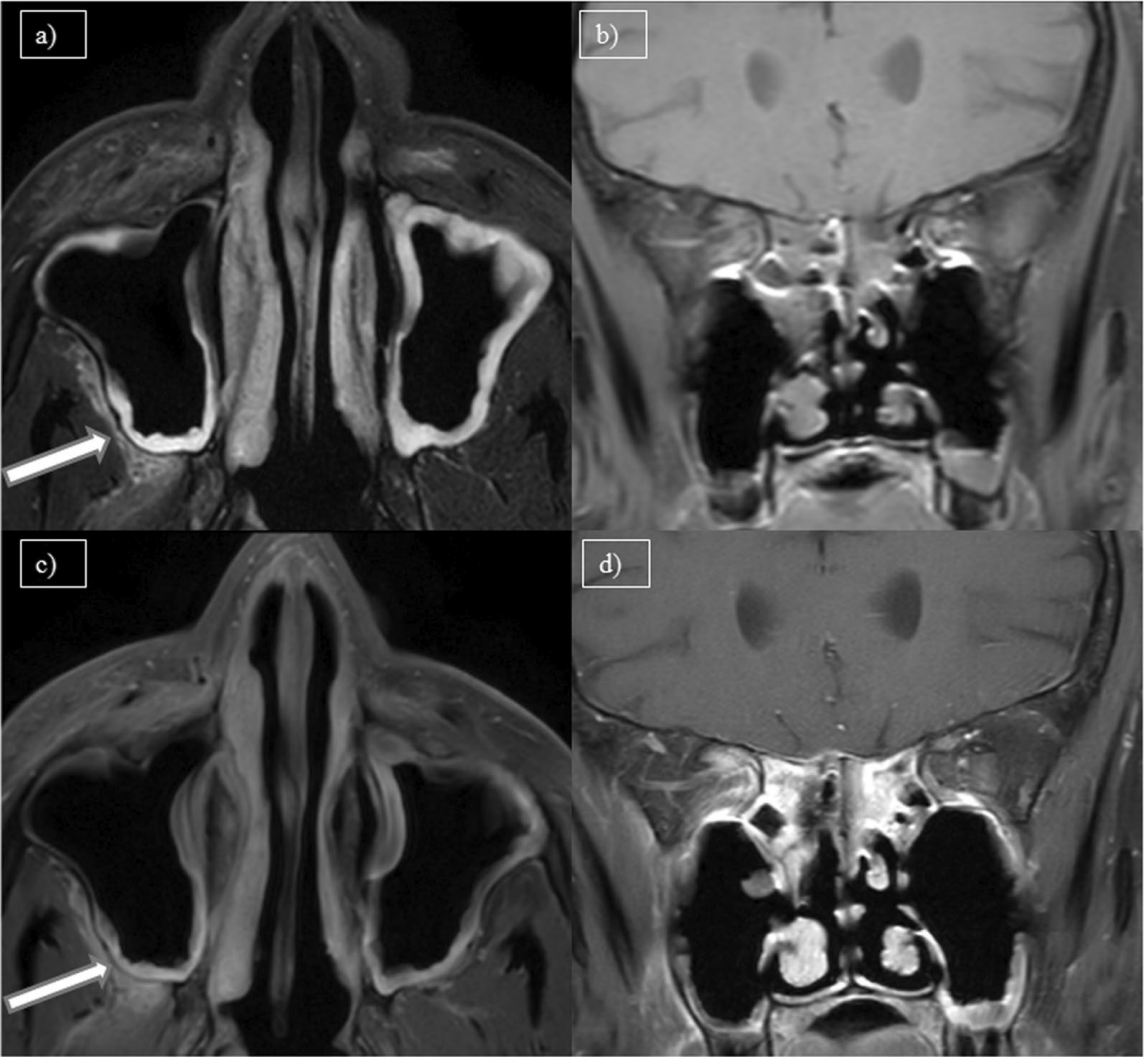
**a** MRI Axial T2W-FS, **b** coronal pre-contrast T1W-FS, **c** and **d** axial and coronal post-contrast T1W-FS images of a 52-year-old female patient with complaints of headache and rhinorrhea showing bilateral maxillary sinusitis with T2 hyperintense periantral space fat stranding which is showing significant post-contrast enhancement (arrow)

Of the cases with rhinosinusitis, 25(34.2%) showed invasive imaging features with periantral fat involvement in all cases and bone erosions in 18 patients (24.6%). Pathological diagnosis of the 25 instances validated the presence of mucormycosis [*n* = 17(68%)] and aspergillosis [*n* = 3(12%)]. The rest of the five cases showed no fungal elements in KOH staining and nonspecific inflammatory features on histopathological examination (HPE). However, one case with no periantral space involvement showed features of mucormycosis on HPE. Hence, a total of 21 patients with fungal etiology were present (28.7%). Out of 18 cases of mucormycosis in this study, periantral space involvement was seen in 17 cases (94.4%). Hence, periantral space involvement was proven to be a highly specific imaging feature in invasive mucormycosis. Figure 1 depicts a 44-year-old patient showing typical CT features of COVID-19 pneumonia in HRCT thorax and bilateral maxillary sinusitis with periantral space involvement. Figure 2 illustrates periantral space involvement in a 52-year-old patient with invasive sinusitis.

**Fig. 3:**
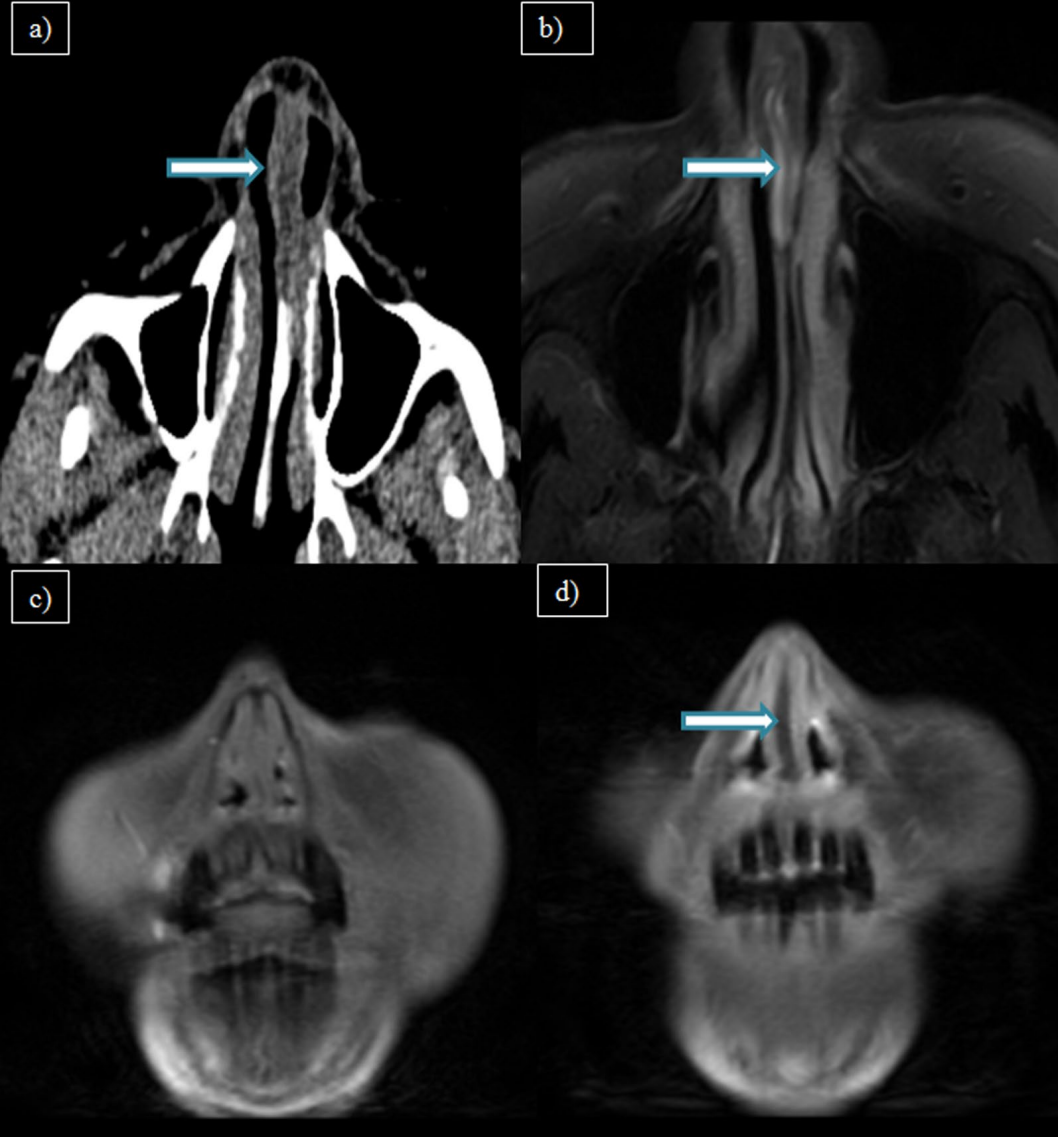
**a** Axial CT paranasal sinus (soft tissue window) of a 68-year-old female patient with complaints of nasal obstruction showing thickened nasal septum with hypodense collection (arrow). **b** MRI axial T2W-FS, **c** coronal pre-contrast T1W-FS and **d** coronal post-contrast T1W-FS images of same patient showing peripherally enhancing collection in the cartilaginous part of nasal septum(arrow)–nasal septal abscess

One case showed nasal septal abscess which on histopathological examination, demonstrated mucormycosis. Figure 3 shows a post-COVID-19 patient with nasal septal abscess.

**Fig. 4:**
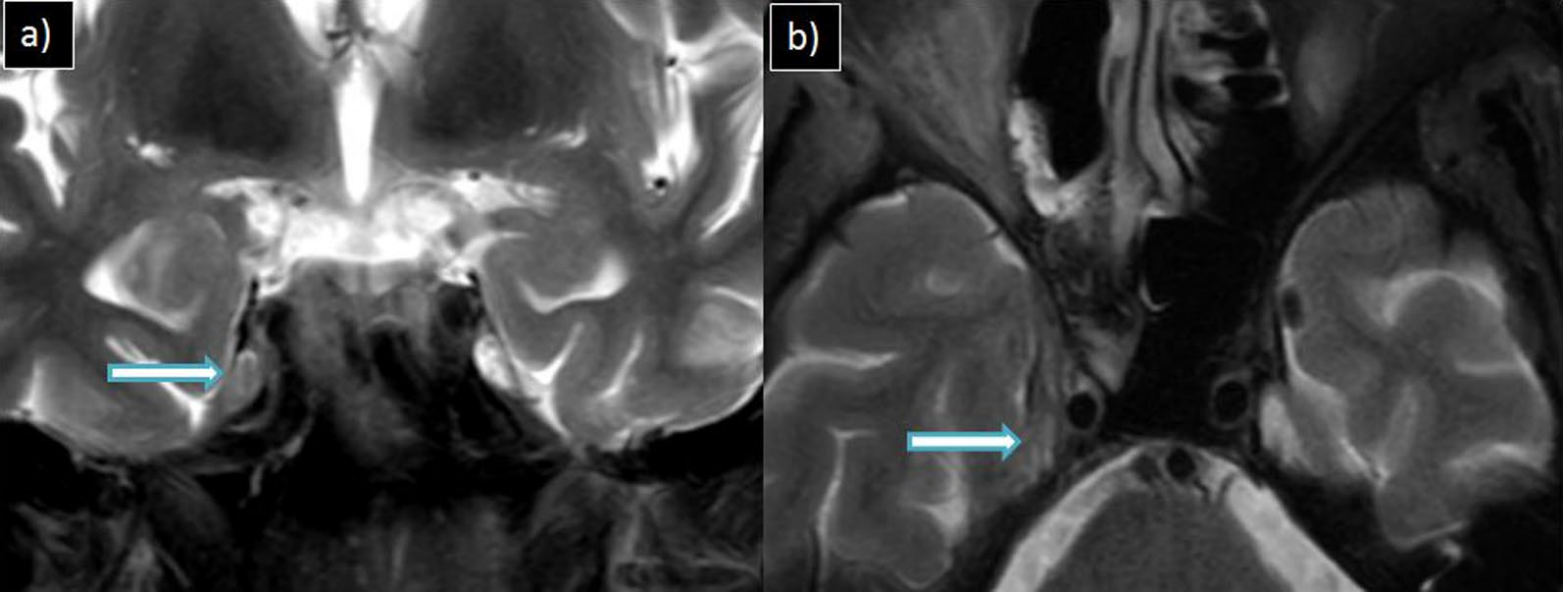
**a** MRI coronal and **b** axial T2W-FS images of a 43-year-old male patient with complaints of rhinorrhea, ocular pain and blurring of vision showing altered signal intensity involving the right Meckel’s cave (arrow)

**Fig. 5:**
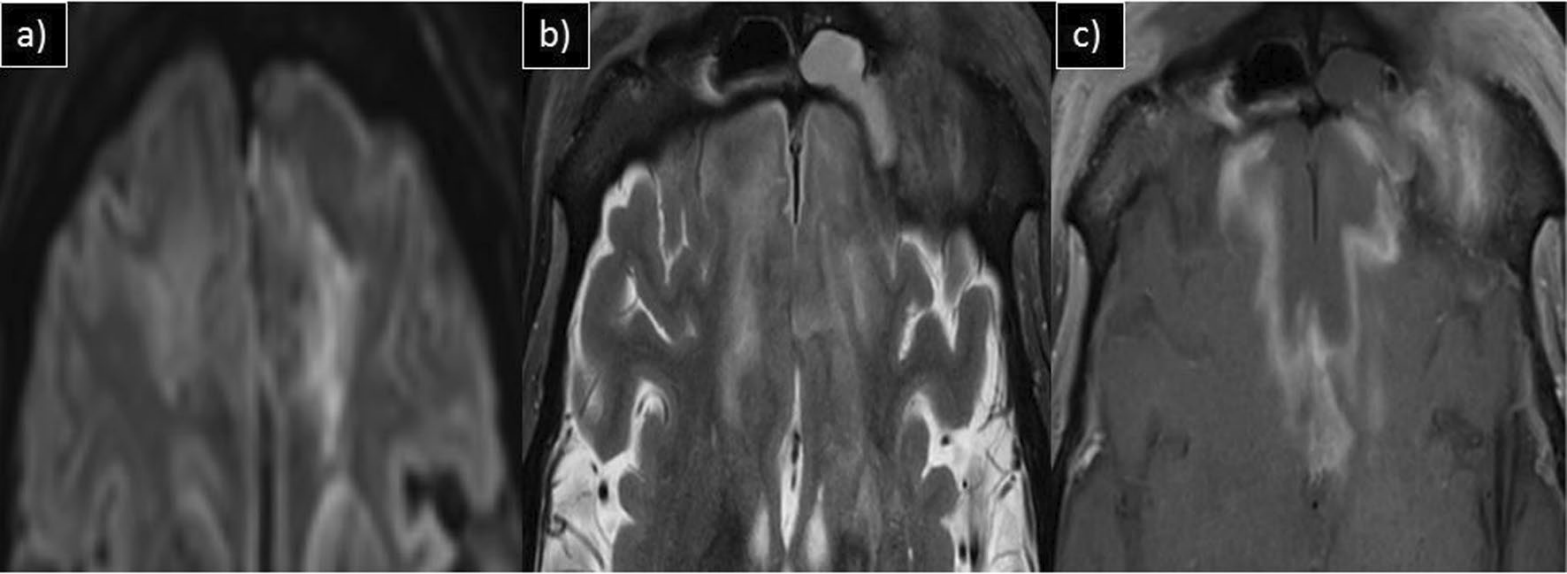
**a** MRI axial iso-DWI, **b** axial T2W-FS and **c** axial post-contrast T1W-FS images of a 37-year-old male patient with complaints of headache and altered sensorium showing features of late cerebritis in bilateral frontal lobe. Left frontal sinusitis is noted

Secondary orbital involvement was seen in 13 cases, intracranial involvement in eight cases and craniofacial osteomyelitis in four cases**.** The various intracranial complications were cerebritis [*n* = 3(37.5%)], subdural empyema [*n* = 3(37.5%)], meningitis [*n* = 6(75%)] and Meckel’s cave involvement [*n* = 1(12.5%)]. Figure 4 depicts Meckel’s cave involvement, and Fig. 5 shows features of late cerebritis in two different patients of invasive sinusitis.

#### Correlation between invasive rhinosinusitis with NLR and comorbidities

As described above, 25 cases (34.2%) showed invasive imaging features among 73 patients of rhinosinusitis. In this subset of rhinosinusitis cases, a statistically significant positive correlation was observed between the presence of imaging features suggestive of invasive etiology and NLR (ρ− 0.590, *p* < 0.001), indicating that with an increase in the NLR, there was an increase in the invasive imaging features with subsequent complications. There was also a significant positive correlation between the imaging features of invasive etiology and comorbidities (ρ− 0.368, *p* = 0.001), indicating that invasive features were more commonly seen in the patients with comorbidities.

There were two deaths among 73 patients, with both patients having invasive imaging features. One case showed secondary orbital complications, and another showed both orbital and intracranial complications. However, there was no statistically significant correlation between the invasive features and mortality (ρ − 0.33, *p* = 0.0484).

#### Orbital manifestations

**Fig. 6:**
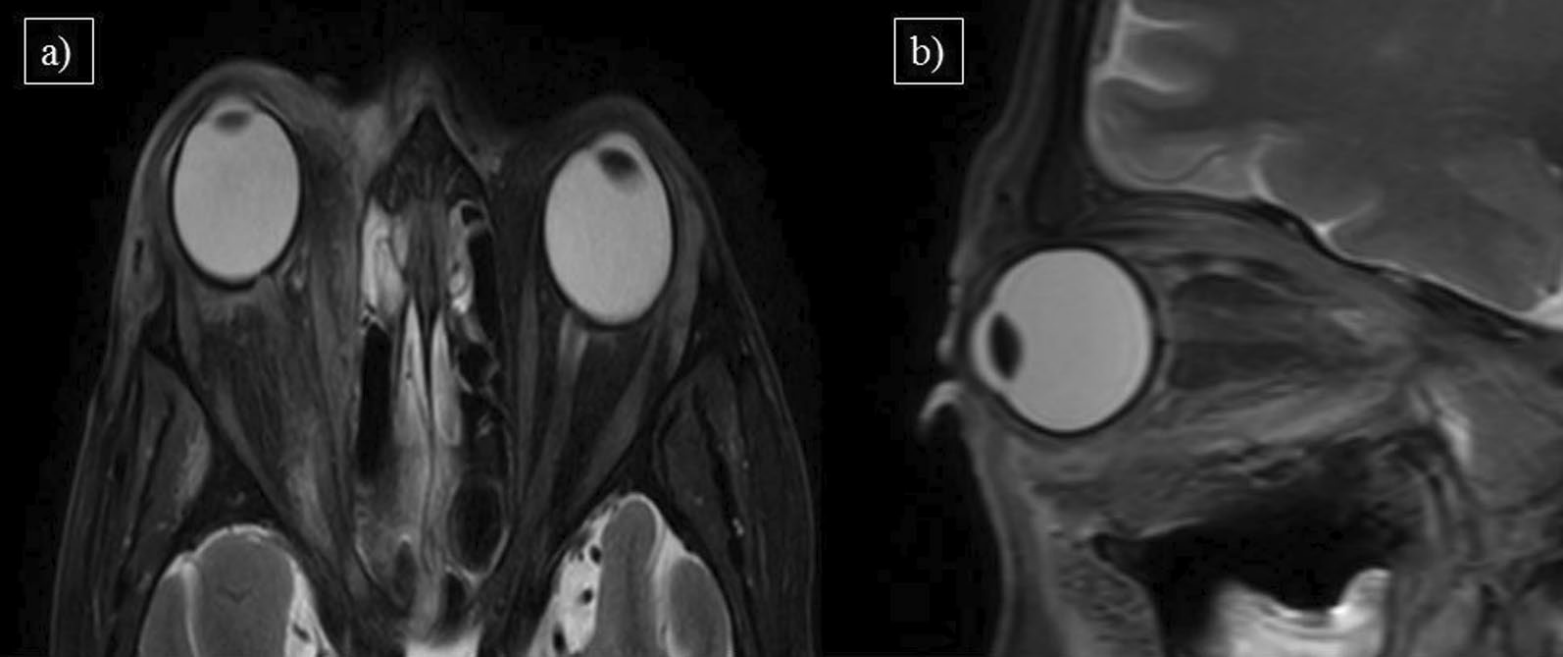
**a** MRI Axial T2W-FS and **b** sagittal T2W-FS images of a 50-year-old male patient with complaints of right sided proptosis and ocular pain showing features of pre and post-septal orbital cellulitis with resultant proptosis

**Fig. 7:**
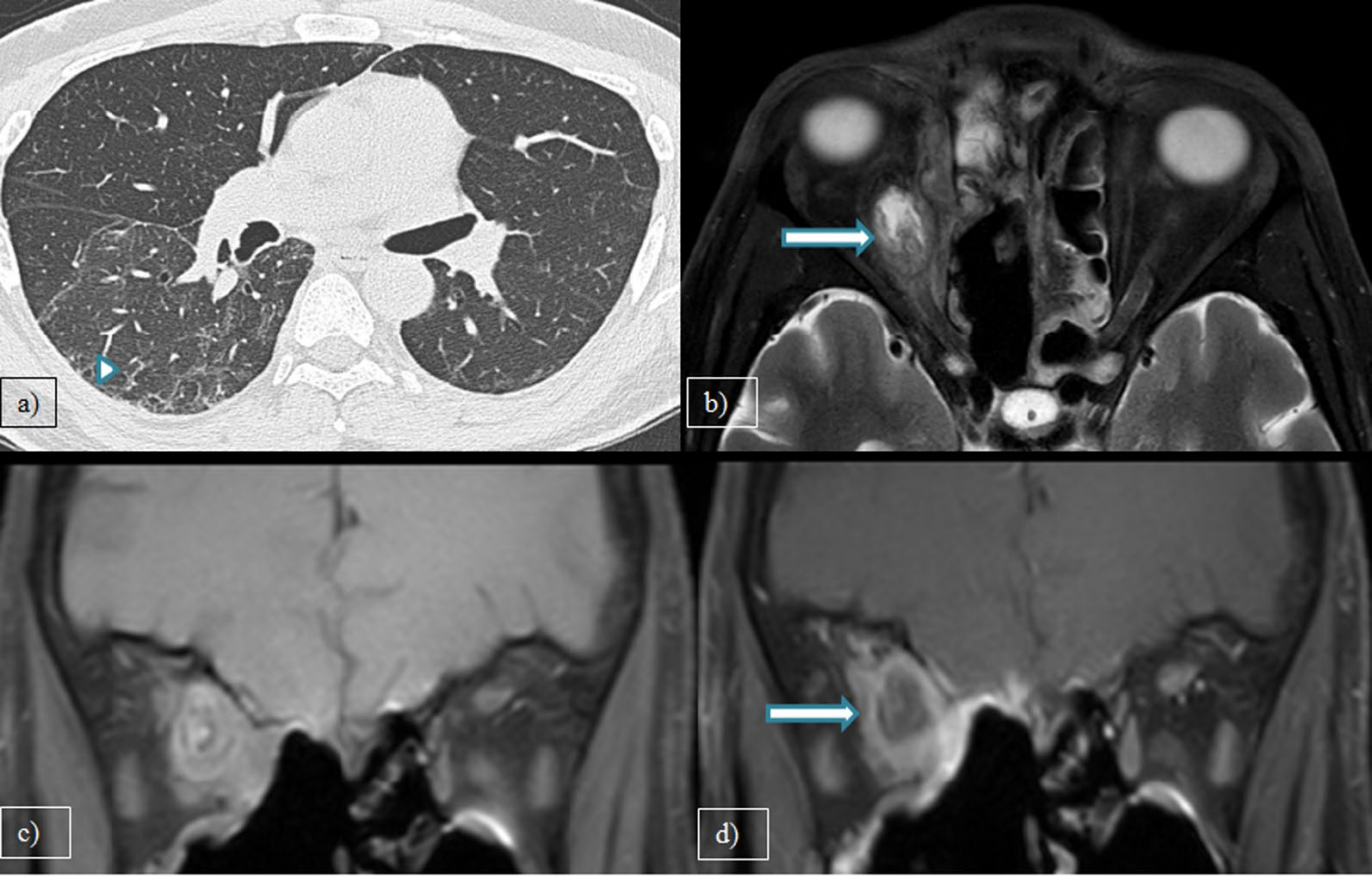
**a** Axial HRCT thorax, **b** MRI Axial T2W-FS, **c** coronal pre-contrast T1W-FS and **d** coronal post-contrast T1W-FS images of a 50 years old male patient with complaints of ophthalmoplegia and ocular pain showing patchy peripheral fibrotic changes with superimposed ground glass opacities (arrowhead) and peripherally enhancing intraconal abscess in the right orbit(arrow). Bilateral ethmoidal sinusitis is also seen

In this study, a total of 14 cases showed orbital involvement (17.9%), with one case diagnosed as dacryoadenitis (primary orbital manifestation), and the remaining 13 cases were secondary to invasive sinusitis (secondary orbital manifestation). Among the secondary orbital manifestations, post-orbital cellulitis was the most common imaging feature [*n* = 13 (92.8%)], followed by pre-septal cellulitis [*n* = 8 (57.1%)], optic neuritis [*n* = 5(35.7%)], orbital apex involvement [*n* = 4(28.5%)], cavernous sinus involvement [*n* = 3(21.4%)] and intraconal abscess [*n* = 2(14.2%)]. The orbital manifestations are depicted in Table 4. Figure 6 shows a case of pre and post-septal orbital cellulitis, and Fig. 7 shows a post-COVID-19 patient with intraconal orbital abscess appearing as T2 hyperintense collection in the right orbit showing peripheral enhancement on the post-contrast study.

**Fig. 8:**
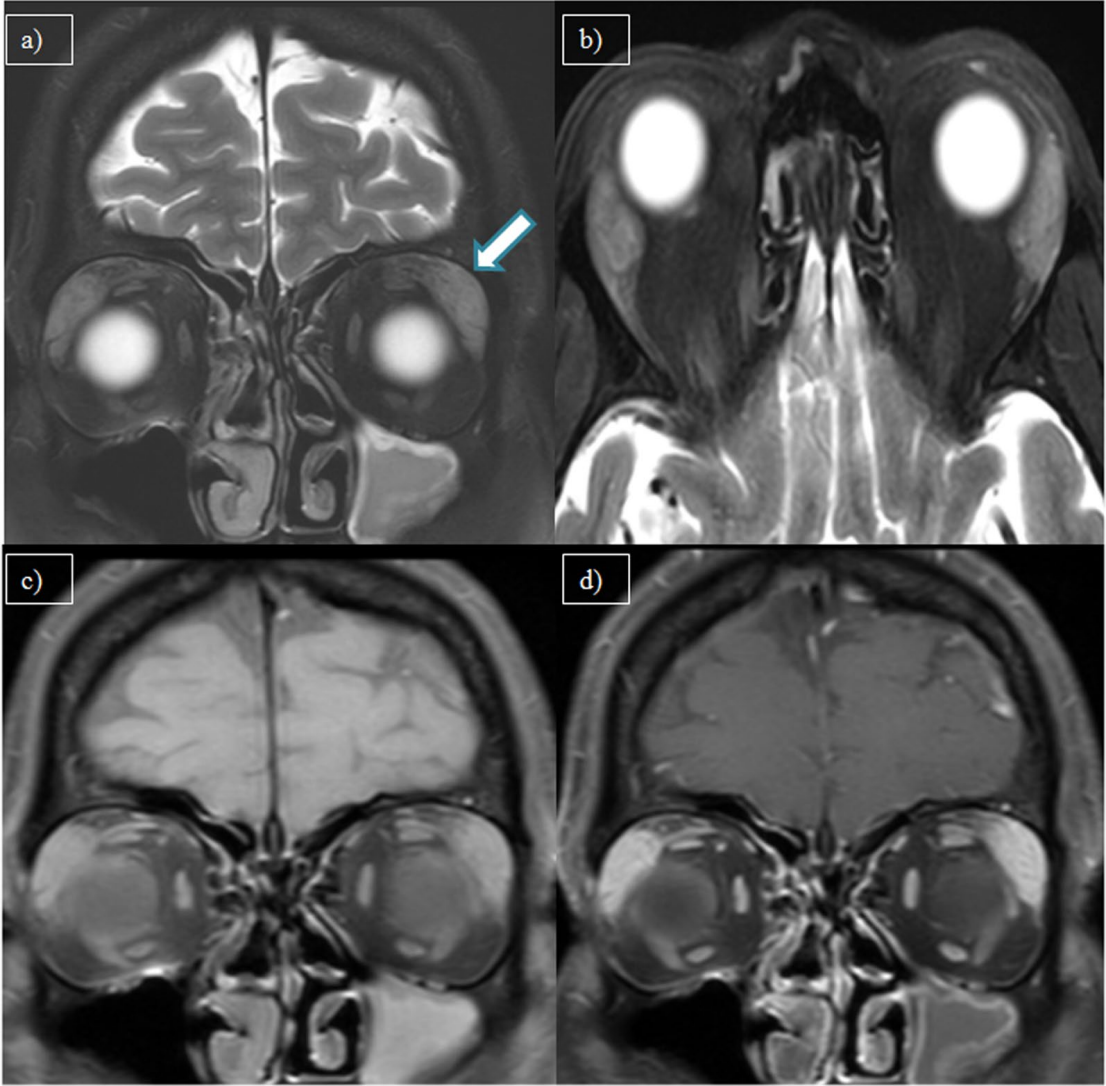
**a** MRI coronal T2W-FS, **b** axial T2W-FS, **c** coronal pre-contrast T1W-FS, and **d** coronal post-contrast T1W-FS images of a 49-year-old male patient with complaints of headache showing bilateral symmetrical enlargement of lacrimal gland appearing hyperintense on T2W (arrow) and showing homogenous post-contrast enhancement—dacryoadenitis

The case of dacryoadenitis in present study was evaluated to rule out autoimmune etiology and other infective etiology before attributing it to viral etiology. No imaging features of tuberculosis and sarcoidosis were found on the HRCT thorax. Anti-nuclear antibodies (ANA), ACE, C-ANCA, P-ANCA and CSF studies were negative. After ruling out other etiologies, it was concluded to be secondary to COVID-19. Figure 8 depicts a case of dacryoadenitis, where bilateral lacrimal glands are diffusely enlargement with homogenous enhancement on post-contrast study. No other orbital inflammatory features were present.

#### Otological manifestations

**Fig. 9:**
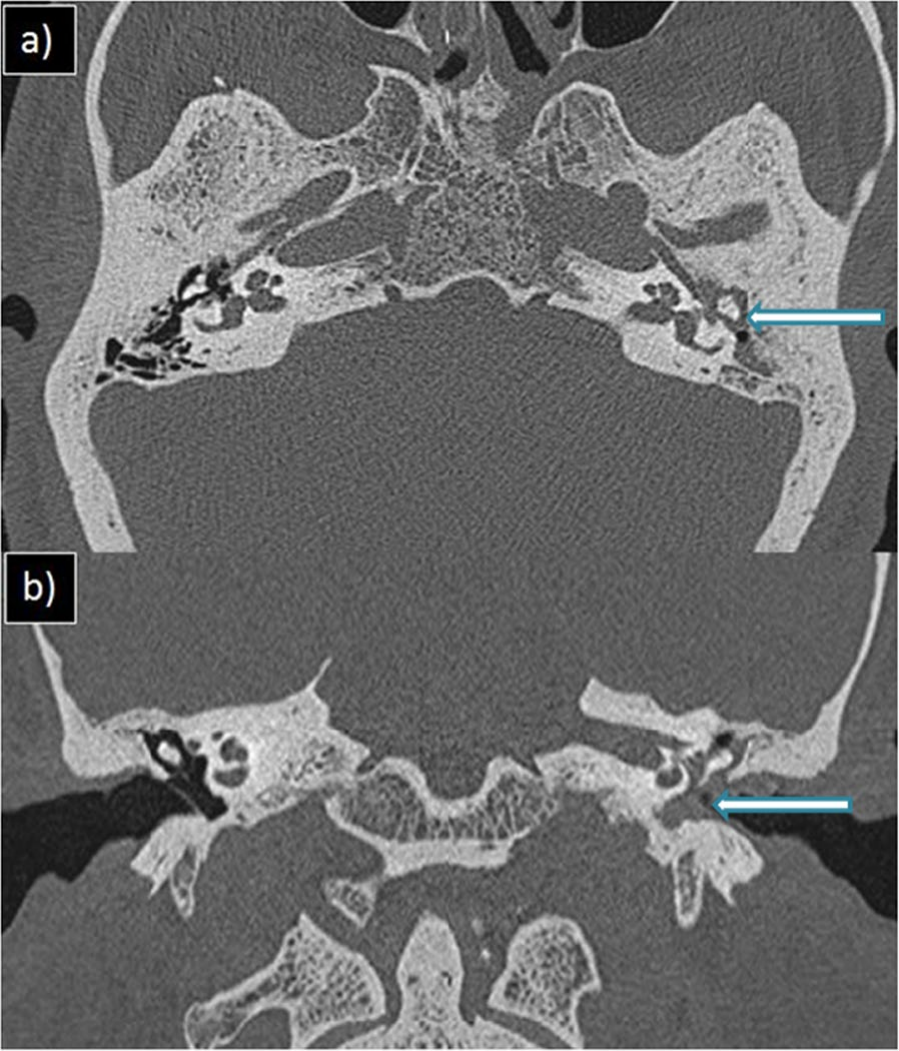
**a** axial and **b** coronal HRCT temporal bone images of 40-year-old female patient of with complaints of otalgia showing soft tissue attenuation in left middle ear and mastoid air cells with no obvious bone/ossicular erosions (arrow)—non-invasive oto-mastoiditis

**Fig. 10:**
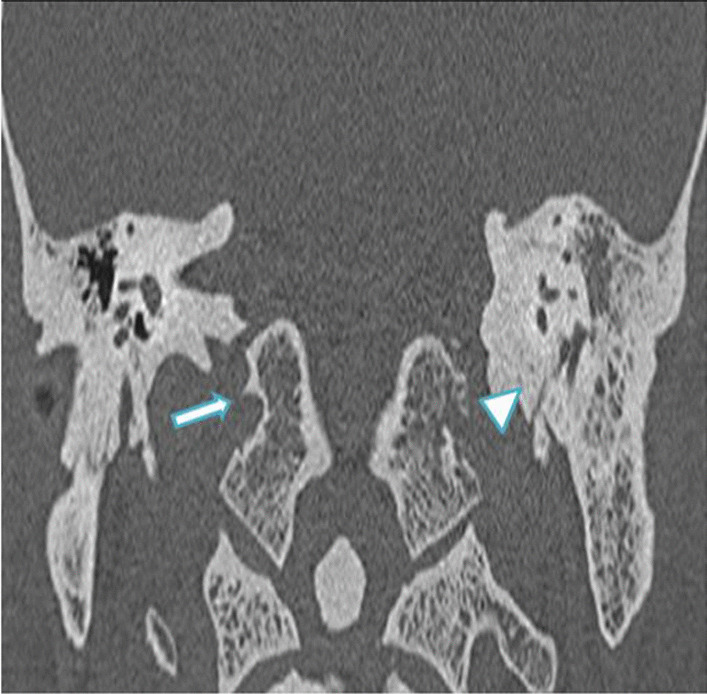
Coronal HRCT temporal bone image of a 59-year-old male patient with invasive oto-mastoiditis and resultant skull base osteomyelitis showing erosions of left hypoglossal canal and jugular tubercle (arrow head). Right hypoglossal canal and jugular tubercle is normal (arrow)

**Fig. 11:**
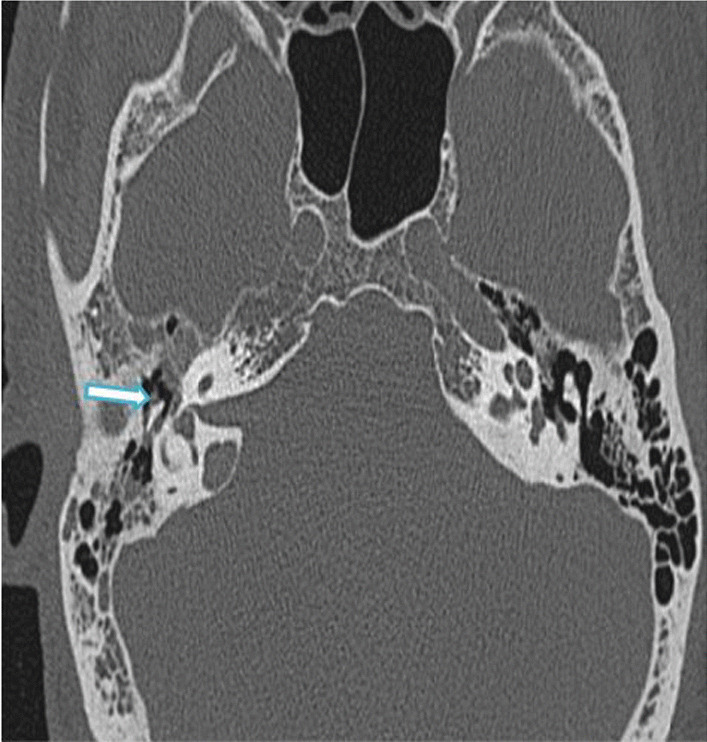
Axial HRCT temporal bone image of a 66-year-old male patient with complaints of otalgia and reduced hearing showing soft tissue attenuation in right middle ear and mastoid air cells with ossicular erosion of head of malleus (arrow)—invasive oto-mastoiditis

In this study, six cases showed otological manifestations (7.6%). Out of these, three cases showed features of otitis media with mastoiditis, and three cases showed isolated mastoiditis. Among the otitis media cases, two showed invasive features with resultant skull base osteomyelitis. On histopathological examination, mucormycosis was isolated from the sample. Figure 9 shows a case of noninvasive oto-mastoiditis. Figure 10 shows a post-COVID-19 case of invasive oto-mastoiditis with skull base osteomyelitis, and Fig. 11 shows a case of invasive oto-mastoiditis with ossicular erosion.

#### Salivary gland involvement

In this study, two cases showed features of parotitis (2.5%). Both cases were COVID-19-recovered patients who presented with pain and swelling over parotid regions. On ultrasonography, both cases showed enlargement and heteroechoic echopattern of bilateral parotid glands with increased internal vascularity. One case showed multiple sub-centimetric intra-parotid lymph nodes. Other salivary glands were normal.

#### Cervical lymphadenopathy

Three (3.8%) cases presented with primary cervical lymphadenopathy. All the cases were young COVID-19-recovered patients who presented with primary symptoms of neck swelling. On the ultrasonography, commonly involved cervical stations were levels IB, II, III and IV. Two cases had a loss of fatty hilum, and others showed intact fatty hilum. Further, US guided FNAC was done, which showed features of reactive lymphadenitis secondary to viral infection.

#### Neck vessels

Two cases showed neck vessel involvement (2.5%). Two young patients with a history of focal neurological deficit were subjected to an MRI brain and neck vessel Doppler. On imaging, one case demonstrated a floating thrombus in the left carotid bulb extending to the proximal internal carotid artery causing ~ 70–80% luminal narrowing, and another case showed complete thrombosis of the left internal jugular vein.

## Discussion

Infection with severe acute respiratory syndrome coronavirus 2(SARS-CoV-2) results in coronavirus disease 2019 (COVID-19) [1]. COVID-19 impacts numerous organ systems with diverse clinico-radiological manifestations, of which pulmonary involvement is known to be the most frequent and severe [1]. Second wave of the pandemic begot an umpteen number of cases demonstrating a wide range of head and neck manifestations in our institute. The study conducted herein explains the same and their complications.

In the cohort, among sino-nasal manifestations, the most common presenting symptoms were headache (68%) and nasal obstruction (61.3%). Among orbital and otological manifestations, proptosis and otalgia were the most common symptoms, respectively. Most of these patients were in the age group between 51–60 and 31–40 years (25.6–23.0%). There was male predilection (66.7%) in this study.

**Fig. 12:**
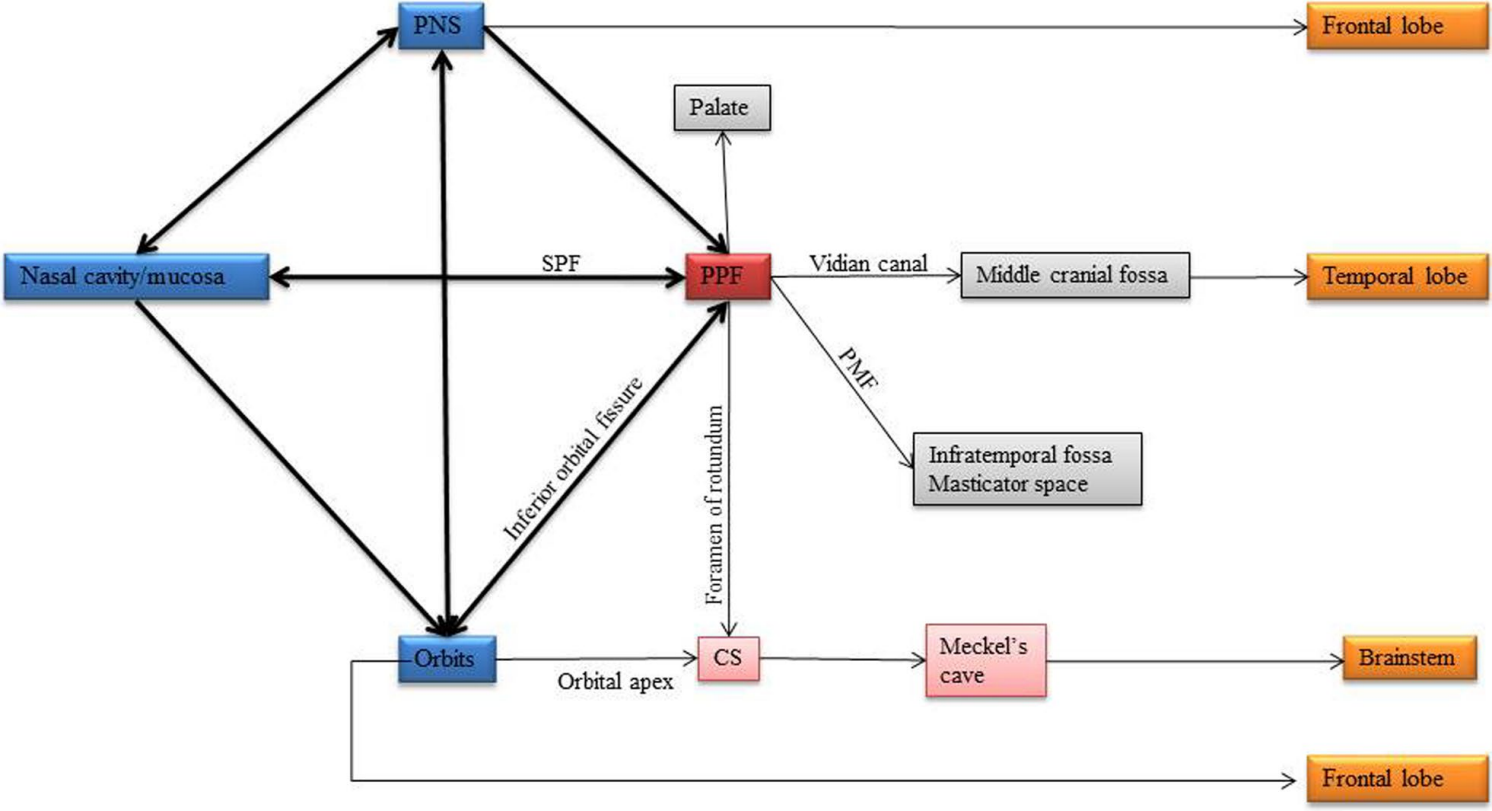
Pathway of spread in invasive rhinosinusitis; *PNS*: paranasal sinus, *SPF*: sphenopalatine foramen, *PPF*: pterygopalatine fossa, *CS*: cavernous sinus, *PMF*: pterygomaxillary fissure

In the present study, rhinosinusitis accounted for 93.6%, with 34.2% showing invasive imaging features. Fungal co-infection was seen in 28.7%. Few possible reasons for the association of mucormycosis and other coinfections in COVID-19 include immunosuppression secondary to decreased CD4^+^and CD8^+^ T cells by COVID-19 infection per se or the extensive use of steroids and broad-spectrum antibiotics in the management of COVID-19 [7]. As the nature of the disease is still not completely unveiled, it cannot be definitively attributed to it being a complication of the disease per se or its management. The potential pathways of spread in cases of invasive sinusitis which were observed are depicted in Fig. 12. In this study, periantral space involvement was a highly specific imaging feature for invasive sinusitis. Hence, early detection of this finding helps in early management and prevents the disease’s spread.

The prevalence of ocular manifestations among COVID-19 patients ranges between 2 and 32% [8]; however, orbital involvement is relatively rare. Dacryoadenitis is inflammation of the lacrimal glands, which is usually caused by infection or autoimmune disease [9]. Viral etiology is the most common infectious cause resulting in acute non-suppurative dacryoadenitis among children and young adults. Viral pathogens producing dacryoadenitis are believed to occur by the spread of the causative organism from the conjunctiva via the lacrimal ductules into the lacrimal gland or direct hematogenous spread [10]. Another hypothesis may be represented by an immunological response targeting the lacrimal gland in the later phase of the COVID-19 infection [11]. In the already available limited literature, only a single case report of dacryoadenitis in COVID-19 has been reported [10]. In addition, the incidence of dacryoadenitis in current study was 1.2%.

The incidence of otological manifestations was 7.6%. Raad et al. [3] suggested that the otological manifestation could be either a manifestation or complication of COVID-19. A few viruses, like coronavirus, respiratory syncytial virus, rhinoviruses, adenovirus, influenza and parainfluenza virus, are known to cause upper respiratory infections and subsequently induce acute otitis media [3]. They can also predispose to bacterial or fungal co-infection. The SARS-CoV-2 virus is found in high concentrations in the nose and nasopharynx, which in turn has communication with the middle ear and mastoid air cells resulting in their secondary involvement. The presence of otitis media should raise suspicion of COVID-19 and look for invasive features and complications [12].

In this study, the incidence of cervical lymphadenopathy as the primary presenting complaint was seen in 3.8%, with commonly involved groups IB, II, III and IV. Distinguin et al. [13] also observed group II (upper jugular group) being the most involved. Pharyngeal structures like tonsils and adenoids are drained by group II lymph nodes. It is believed that the COVID-19 virus causes inflammation of the nasopharynx and oropharynx, causing local immune reactions resulting in cervical lymphadenopathies [13].

The incidence of parotitis was 2.5% in this study. On ultrasonography, we observed diffuse enlargement of bilateral parotids with heterogeneous echopattern and increased vascularity. One case showed multiple intra-parotid lymph nodes. These imaging features were consistent with a few case reports demonstrating the occurrence of acute parotitis related to COVID-19 [14, 15]. The possible theoretical cause of parotitis in COVID-19 might be secondary to the direct spread of SARS-CoV-2 to the parotid glands due to the presence of virus receptor angiotensin-converting enzyme 2(ACE-2).The other hypothesis is intra-parotid lymphadenitis as a casual factor resulting in secondary parotid inflammation [14]. Hence, we emphasize the need to consider COVID-19 among the differentials for parotitis.

The incidence of neck vessel involvement in the present study was 2.5%. COVID-19 is known to be highly pro-thrombotic, resulting in alterations of the coagulation cascade due to the abundant presence of virus ACE-2 receptors in the vascular endothelium [16]. Increased morbidity and mortality are associated with the coagulation disorders of COVID-19 [17, 18].

Few limitations have been noted in the study. As this was conducted in a single tertiary referral center, we received a limited number of patients, thus explaining the small sample size. The other limitations were the non-inclusion of CT severity score and vaccination status of the patients; because limited patients underwent HRCT thorax, and only some were vaccinated at the time of the study. We plan further studies on a larger cohort to validate the etiopathogenesis and compare them among vaccinated vs. non-vaccinated groups shortly.

## Conclusions

The high specificity of findings indicating invasive sinusitis and its spread along with its correlation with NLR and presence of co-existing comorbidities, as demonstrated in this study, are vital tools to facilitate & enhance diagnostic ability of radiologists. In the wake of the COVID-19 pandemic, we must note that this pathology can have a large myriad of presentations making it imperative to alert ourselves of its varied head and neck manifestation.

## Data Availability

The datasets generated and/or analyzed during the current study are not publicly available due to privacy of the study participants.

## Abbreviations

COVID-19: Coronavirus disease 2019
NLR: Neutrophil–lymphocyte ratio
SARS-CoV-2: Severe acute respiratory syndrome coronavirus 2
RT-PCR: Reverse-transcriptase polymerase chain reaction
CT: Computed tomography
MRI: Magnetic resonance imaging
PACS: Picture archiving and communication systems
MDCT: Multidetector computed tomography
HRCT: High-resolution computed tomography
DWI: Diffusion-weighted imaging
FLAIR: Fluid-attenuated inversion recovery
GRE: Gradient recalled echo
KOH: Potassium hydroxide
HPE: Histopathological examination
ANA: Anti-nuclear antibodies
ACE: Angiotensin-converting enzyme
ANCA: Antineutrophil cytoplasmic antibodies
CSF: Cerebrospinal fluid
FNAC: Fine needle aspiration cytology
